# Source regions of ragweed pollen arriving in south-western Poland and the influence of meteorological data on the HYSPLIT model results

**DOI:** 10.1007/s10453-017-9471-9

**Published:** 2017-01-20

**Authors:** Daria Bilińska, Carsten Ambelas Skjøth, Małgorzata Werner, Maciej Kryza, Małgorzata Malkiewicz, Justyna Krynicka, Anetta Drzeniecka-Osiadacz

**Affiliations:** 1 Department of Climatology and Atmosphere Protection, University of Wrocław, Wrocław, Poland; 2 National Pollen and Aerobiology Research Unit, Institute of Science and the Environment, University of Worcester, Henwick Grove, WR2 6AJ Worcester, UK; 3 Institute of Geological Sciences, University of Wrocław, Wrocław, Poland

**Keywords:** *Ambrosia artemisiifolia*, Aeroallergens, Back trajectory analysis, HYSPLIT

## Abstract

We have investigated the relationship between the inflow of air masses and the ragweed pollen concentration in SW Poland (Wrocław) for a 10-year period of 2005–2014. The HYSPLIT trajectory model was used to verify whether episodes of high concentrations can be related to regions outside of the main known ragweed centres in Europe, like Pannonian Plain, northern Italy and Ukraine. Furthermore, we used two different meteorological data sets (the global GDAS data set and from the WRF mesoscale model; the meteorological parameters were: U and V wind components, temperature and relative humidity) into HYSPLIT to evaluate the influence of meteorological input on calculated trajectories for high concentration ragweed episodes. The results show that the episodes of high pollen concentration (above 20 pm^−3^) represent a great part of total recorded ragweed pollen in Wrocław, but occur rarely and not in all years. High pollen episodes are connected with air masses coming from south and south-west Europe, which confirms the existence of expected ragweed centres but showed that other centres near Wrocław are not present. The HYSPLIT simulations with two different meteorological inputs indicated that footprint studies on ragweed benefit from a higher resolution meteorological data sets.

**Electronic supplementary material:**

The online version of this article (doi:10.1007/s10453-017-9471-9) contains supplementary material, which is available to authorized users.

## Introduction

*Ambrosia artemisiifolia* (common ragweed) is a wind pollinating annual plant that belongs to the Asteraceae family (Fumanal et al. 2007; Smith et al. 2013). *Ambrosia artemisiifolia* is an invasive species for Europe [among *Ambrosia* genus only *A. maritima L*. is native (Makra et al. 2005)]. Ambrosia’s pollen is becoming a serious problem in Europe, because there is an ongoing spreading of ragweed in Europe and a number of countries have already or are deriving mitigation plans against ragweed (Hamaoui-Laguel et al. 2015). The number of areas occupied by ragweed in Europe is increasing probably due to the climate changes and the occurrence of areas favourable to ragweed growth (Jäger 1991; Fernández-Llamazares et al. 2012; Hamaoui-Laguel et al. 2015). This means that there is a constant need for updated information on major ragweed areas and methods for detecting new invasion fronts.

*Ambrosia artemisiifolia* is a very troublesome plant because its pollen is very allergenic (Burbach et al. 2009). Very low concentrations such as 5–10 pm^−3^ can cause allergic reactions in sensitised patients, and the symptoms include rhino-conjunctivitis and more rarely contact dermatitis or urticaria (Taramarcaz et al. 2005). Concentrations of 10–20 pm^−3^ nearly always cause allergic symptoms in these patients (Bergmann et al. 2008). *Ambrosia* pollen can induce asthma twice as likely than other (Skjøth et al. 2010). There is an increasing trend in sensitisation in Europe, and large geographical variation in sensitisation rates is observed among the allergic population (Burbach et al. 2009). The highest concentrations of common ragweed pollen in Europe are observed in the Pannonian Plain (Járai-Komlódi 2000; Rybníček et al. 2000; Mosyakin and Yavorska 2002; Peternel et al. 2005; Šikoparija et al. 2006), the northern part of Italy (Zanon et al. 2002; Cecchi et al. 2006), and central and south-east France (Laaidi and Laaidi 1999; Laaidi et al. 2003). These source areas regularly affect other parts of Europe with airborne ragweed pollen being transported for long distances, and Poland appears to be in particular exposed to episodes of airborne common ragweed pollen from other regions (Smith et al. 2008; Kasprzyk et al. 2011; Šikoparija et al. 2013).

Three species of *Ambrosia* genus have been found in Poland: *Ambrosia artemisiifolia*, *Ambrosia psilostachya*, *Ambrosia trifida* (Mirek et al. 2002; Chłopek et al. 2011). Almost the whole area of Poland is suitable to spread and establishment of *A. artemisiifolia* (Karnowski 2001; Tokarska-Guzik et al. 2011). Common ragweed is found predominantly in south-western part of Poland (Tokarska-Guzik et al. 2011). According to Tokarska-Guzik et al. (2011) in 1892, common ragweed was found in Wrocław, and its spread was confirmed between 1951 and 2009. In present, the presence of common ragweed in Wrocław is not reported in the literature. The occurrence of *A. artemisiifolia* is confirmed in the Silesian Uplands (e.g. in Żory town, along the road connecting Katowice and Cieszyn) (Tokarska-Guzik et al. 2011). However, the high pollen concentrations are observed in Wrocław and the source regions can be determined by the atmospheric transport models. One of the most commonly applied models in aerobiology is HYSPLIT that has been used to study atmospheric transport of a number of species such as *Olea* and *Quercus* pollen in southern Iberian Peninsula (Hernandez-Ceballos et al. 2011; Fernandez-Rodriguez et al. 2014; Hernandez-Ceballos et al. 2014), ragweed pollen in several European countries (Stach et al. 2007; Smith et al. 2008; Kasprzyk et al. 2011; Šikoparija et al. 2013), birch pollen in central and northern European countries (Skjøth et al. 2007; Veriankaite et al. 2010). However, the HYSPLIT model is sensitive to the meteorological input data (Hernandez-Ceballos et al. 2014) in particular in areas with complex terrain. The major *A. artemisiifolia* centres in France, the Pannonian Plain and northern Italy are all surrounded by the mountains, which are factor determining spreading of *Ambrosia* pollen. Southern Poland is also bordered by Sudetes and Carpathian Mountains; thus, the long-range transport of *Ambrosia* pollen to Poland is associated with the move of air masses over such areas. According to Smith et al. (2008), long-distance transport over the mountains is dependent on their height and the depth of the PBLs. Deep PBL is usually connected with large surface heat fluxes and convection, which allows distribution of pollen in the entire PBL. Additionally, if pollen grains are found in the top of the PBLs, their settling can take a few days and pollen can be carried long distances with air masses. If the PBL is deep, and mountains are lower than the PBLs, the transport over the mountains occurs. In case of Carpathian Mountains (at least their central part) transport of pollen grains is limited because of mountains height, which is often similar or higher than the depth of PBLs.

The problem of a long-range transport of *Ambrosia* pollen in Poland has been described in such cities like Poznań, Gdańsk, Szczecin, Sosnowiec, Zagórów, Łódź, Rzeszów, Kraków (Stach et al. 2007; Smith et al. 2008; Kasprzyk et al. 2011; Šikoparija et al. 2013). Smith et al. (2008) examined temporal variation and back trajectories during the 7–10th of September 2005. The research taken by Stach et al. (2007) and also by Šikoparija et al. (2013) in Poznań has shown the possibility of carrying *Ambrosia* pollen grains by long-distance transport. It was also confirmed in research conducted by Kasprzyk et al. (2011).

The main aim of our study was to investigate the relationship between the inflow of air masses and the concentration of airborne ragweed pollen in south-west Poland for a 10-year period of 2005–2014. We investigated whether high concentrations of airborne ragweed pollen could be attributed to regions outside of the main known centres in Europe. Finally, we explored whether our results could be affected by the meteorological input into the HYSPLIT trajectory model by using two different data sets for specific episode studies: the standard data set with HYSPLIT–GDAS, with 1° spatial resolution and a high-resolution data set obtained from the WRF model, with a spatial resolution of 12 km × 12 km over central Europe. Up to our best knowledge, the study like this has never been taken before for this location and that long period.

## Materials and methods

### Pollen data

The daily airborne ragweed pollen concentration data cover a 10-year period of 2005–2014. The measurements were taken at the Wrocław station (630,000 citizens, valid for 2011, GUS Wrocław, http://stat.gov.pl/) in south-west Poland (51.1164 N, 17.0278 E) using a Burkard 7-day volumetric pollen trap. The sampler is placed in the city centre, on the roof of the building at a height of 20 m above ground level. In the vicinity of the sampling site, there are a dense urban built-up areas and scanty patches of greenery. From the south, the building is surrounded by an alley of plane trees, while several horse-chestnut trees and small birches grow to the north of the building (Malkiewicz et al. 2014).

Slides with airborne pollen were analysed following the recommendations of the International Association for Aerobiology (Galán et al. 2014). Pollen grains were counted under a light microscope with 400 magnification along 4 longitudinal transects. The results were expressed as the number of pollen grains per cubic metre of air as a daily mean value (pm^−3^) (Malkiewicz et al. 2014). Data were produced regularly and kept in a secure monitoring data base.

We analysed pollen concentration for 2 months (August and September) of each year during the 2005–2014, which cover the blooming season of *Ambrosia artemisiifolia* in Pannonian Plain (e.g. Makra et al. 2005; Šikoparija et al. 2009). The data were divided into two groups of low (≤20 pm^−3^) and high (>20 pm^−3^) airborne ragweed pollen concentration (in short hereinafter “low” and “high” concentrations). This approach has been also used in previous studies, (Stach et al. 2007; Šikoparija et al. 2009; Kasprzyk et al. 2011), which is associated with the fact, that concentrations of ragweed pollen above 20 pm^−3^ nearly always cause allergic symptoms in sensitive patients (Bergmann et al. 2008). The low and high groups of airborne pollen concentration periods were analysed separately and compared to the entire data set. The 2-month sum of the daily concentration used in this paper is the sum of the daily average airborne ragweed pollen concentrations during 2 months—August and September for each investigated year.

The most severe episodes of high ragweed concentrations have been chosen from the whole investigated period. The severe episodes have been defined as days with airborne ragweed pollen concentrations above 20 pm^−3^, occurring for several consecutive days (there were three episodes in 2005–2014, lasting from 4 to 7 days), and for these episodes, hourly concentrations of ragweed were analysed.

Finally, back trajectory analyses (Sect. 3.3) were done:

1. separately for the low, high and all (without division into high or low) airborne pollen concentrations—for August and September of the entire 10-year investigated period (2005–2014),
2. separately for the high and all concentrations of pollen in the air—for August and September for each single year of the 2005–2014,
3. for three selected episodes of high airborne ragweed pollen concentration (5 days in 2005, 7 days in 2006 and 4 days in 2014).

The footprint area of air masses calculated with HYSPLIT shows only the direction of moving air masses but not the source area of pollen. The combination of direction of air masses inflow and the known sources of *Ambrosia* pollen can together indicate the possible source of ragweed pollen that occurs in Poland.

### The WRF model simulations

The WRF model, version 3.5 (Skamarock et al. 2008), was used for detailed analyses of the selected episodes of high ragweed pollen concentrations in the air. WRF provides both meteorological data that can be used to describe the synoptic situation and data for detailed calculations with the HYSPLIT model. The GFS FNL global analyses were used to define the initial and boundary meteorological conditions for the WRF model. GFS FNL are created and maintained by the National Centres for Environment Prediction (NCEP), with a spatial resolution of 1° × 1° degrees and a vertical resolution of 27 pressure levels. The main physical options in WRF used in this study include the Noah Land Surface Model, YSU boundary layer physics, Dudhia scheme for shortwave radiation and rapid transfer model (RRTM) for longwave radiation, Grell 3D parameterisation with radiative feedback and shallow convection and the Lin microphysics scheme, respectively. The model setup included two nested domains, where the mother domain (d1) covered the entire Europe, with a resolution of 36 km × 36 km and the inner domain (d2) with a grid resolution of 12 km × 12 km covered central Europe and some parts of northern, western and southern Europe. The second domain (Fig. S1) covered the three main European ragweed centres that are identified as the Pannonian Plain, northern Italy and the Rhone valley (France). We have adjusted the vertical resolution in WRF by decreasing the thickness of the lowest layer from 53 to 20 m and doubling the number of layers within the first 1015 m, which gives 48 layers in total. This approach is especially relevant when meteorological output is used to feed chemical transport models, as it has previously been proved that chemical models general benefit with a high number of layers in PBL (Zhang et al. 2010). The data were converted to ARL format, but with 1-h temporal and 12-km spatial resolution as input data for HYSPLIT. Using the WRF data in HYSPLIT, we created three maps (one for each episode described in details in the following section) showing spatial distribution of the trajectories. Additionally, the WRF simulations were used for a synoptic analysis of the prevailing meteorological conditions as WRF provides a full 3D data set of hourly meteorological variables. Selected maps are available as a supplementary material.

### Backward trajectories in HYSPLIT

The HYSPLIT (HYbrid Single-Particle Lagrangian Integrated Trajectory) model is designed for a quick response to atmospheric emergencies, diagnostic case studies, or climatological analyses using previously gridded meteorological data. It consists of a modular library structure with main programs for each primary application: trajectories and air concentrations (Draxler et al. 2014). Here the HYSPLIT model was used to analyse the entire observational record (2005–2014) with the meteorological data obtained from the Global Data Assimilation System (GDAS) with a 1° × 1° spatial resolution and 3-h temporal resolution. We used 96-h backward trajectories with 2-h intervals and with three different altitudes: 500, 1000 and 1500 m above ground level (agl) as this is the standard methodology in many aerobiological studies (Fernández-Rodríguez 2014; Hernandez-Ceballos et al. 2014; de Weger et al. 2016) These parameters were taken in order to observe relationships between different inflowing air masses and concentration of ragweed pollen in the air. Trajectories were calculated for Wrocław (51.1054N 17.0890E) and matched with the corresponding records with measured ragweed pollen concentrations. The trajectories were then sorted into two groups—days with low and high concentrations of airborne pollen according to Stach et al. (2007). Each day was represented by 36 trajectories, which reduced the uncertainty associated with individual trajectories (Stach et al. 2007) and also took into account the variation in air flow pattern during the day. Using the HYSPLIT trajectories, footprint maps were created using the two main groups of airborne pollen concentration as in Skjøth et al. (2015). The footprints area of air masses was gridded on a regular grid to show the total number of trajectories crossing each grid cell. The analysis was done for the entire investigated period (as a mean value) and individually for each year of the period.

Three the most severe episodes with high concentrations of airborne ragweed pollen were analysed in detail. This was done with the HYSPLIT model and two different, in terms of, e.g. spatial resolution, meteorological data sets—one from GDAS and second from the 12 km × 12 km WRF model. Application of two different meteorological data for the episodes enabled to verify the influence of spatial resolution of input data on the results.

## Results

### Airborne ragweed pollen occurrence in 2005–2014

**Fig. 1:**
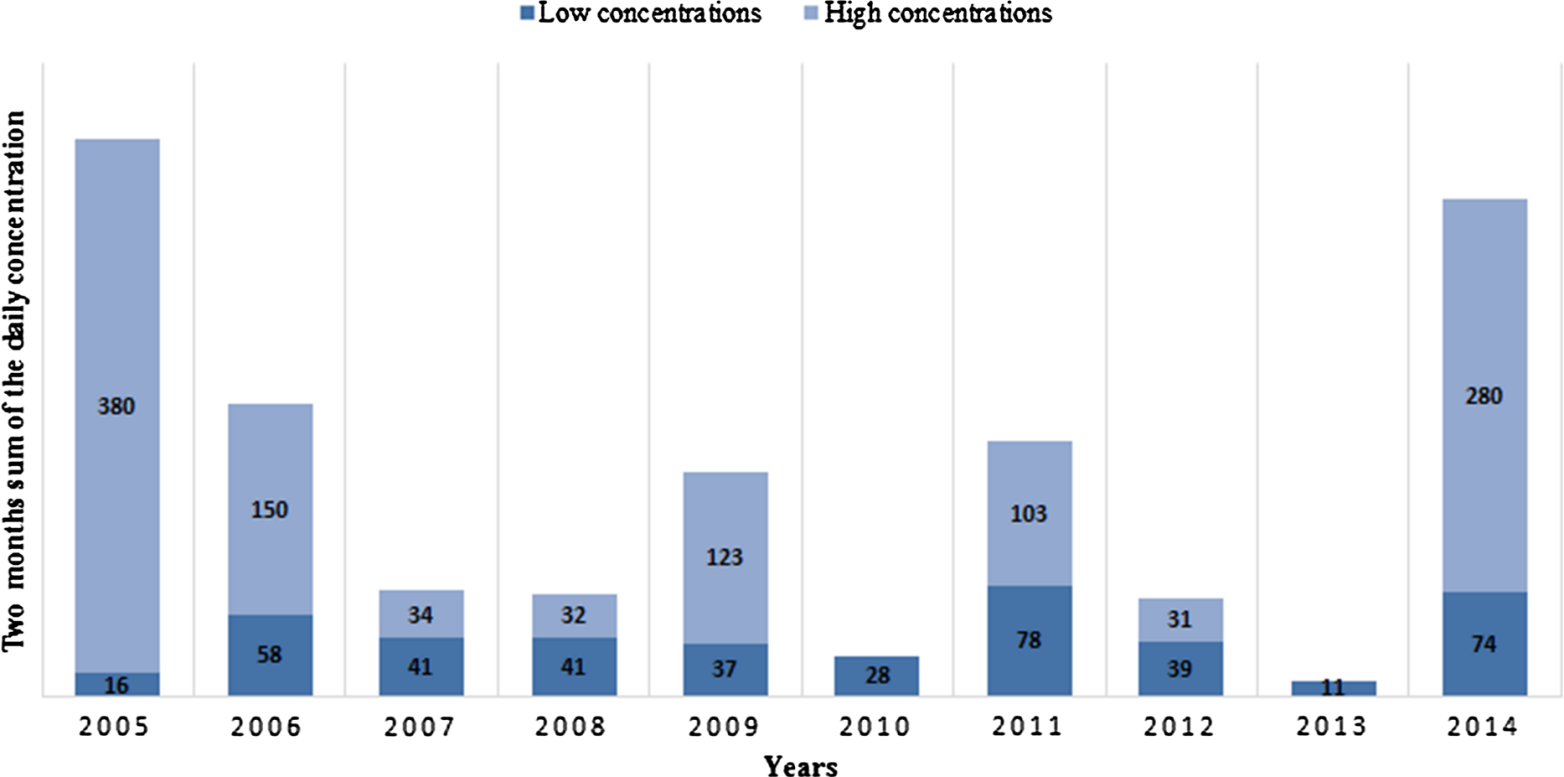
2-month sum of the daily concentration of airborne ragweed pollen in Wrocław station divided into low (≤20 pm^−3^) and high (>20 pm^−3^) values

The 2-month sum of the daily concentrations of airborne ragweed pollen in Wrocław for 2005–2014 varies significantly and ranges from 11 grains in 2013 to 380 grains per m^3^ in 2005 (Fig. 1). Pollen grains from the high airborne pollen concentration episodes represent a great part of total recorded *Ambrosia* pollen in Wrocław. This is particularly evident for the years with the highest annual sums of grains (2005 and 2014). For those years, the contribution of sum of grains for days with high airborne pollen concentration was 96% (5 days) and 76% (5 days) of the total sums of grains. There are only 2 years (2010 and 2013), in which high airborne pollen concentrations were not observed.

Three episodes of high concentrations of airborne ragweed pollen were observed in Wrocław in 2005–2014. These episodes ranged from 4 to 7 days (16 episode days in total). The highest daily mean pollen concentration during the episodes reached 137 pm^−3^ which was recorded on 6th of September 2005. Additionally, there were 8 individual days in the analysed period with ragweed pollen concentration up to 80 pm^−3^.

### Pollen transport in 2005–2014

**Fig. 2:**
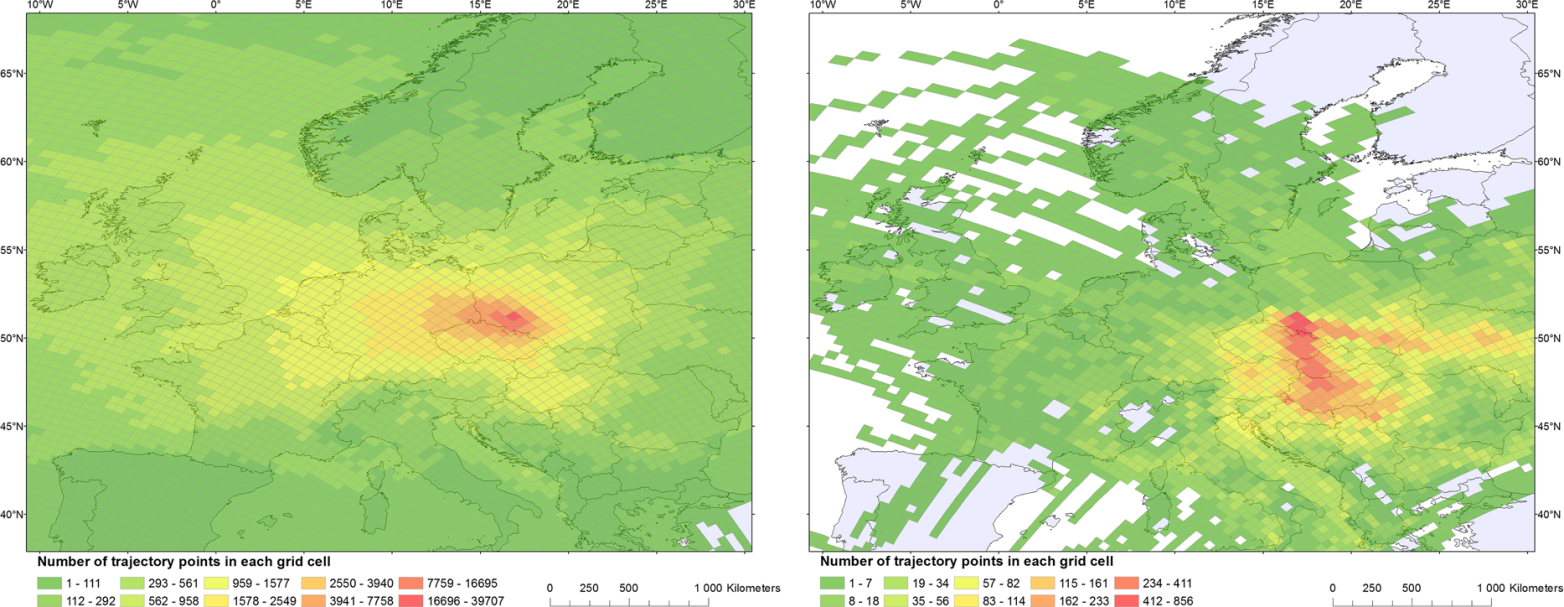
Number of trajectory crossing each grid cell for August and September for all (*left*) and high values (*right*) of ragweed concentrations for years 2005–2014. Calculations are based on HYSPLIT using GDAS data and 96-h back trajectories for Wrocław at 500, 1000 and 1500 m

The total number of trajectories calculated with HYSPLIT is 21,960 (equivalent to 610 days). This covers days both with low and high ragweed pollen concentration in Wrocław. The foot print area of air masses shows that the flow of air masses during the pollen seasons in the entire investigated period (without dividing into daily mean low or high concentration of pollen) was generally originating from western and north-western part of Europe (Fig. 2) and a smaller contribution of air masses originated from southern directions, e.g. Slovakia and Hungary. The number of cases with the flow from the east and south-east is low.

The number of trajectories on days with high *Ambrosia* airborne pollen concentrations is 756 (21 days, Fig. 2). The dominant flow on those days was from south and south-eastern directions. The air masses crossed the Pannonian Plain (the Czech Republic, Slovakia, Hungary) and Ukraine on the high days.

The number of trajectories on days with low *Ambrosia* pollen concentrations is 21,204 (589 days). The foot print area for the daily mean low concentration (≤20 pm^−3^) days was north-western part of Europe. For very few cases, the air masses were of easterly or southerly origin during low days.

### Pollen transport in subsequent years

There is a dominating western and north-western flow during all days in August–September of each individual year of 2005–2014 (Fig. S2). For some years, the figure with the footprint area of air masses shows the second area of high frequency of back trajectories. It is southern or south-eastern direction for years 2006, 2007, 2011, 2014 and eastern direction for 2005, 2008 and 2012. High pollen concentrations in the air are mainly related to the flow either from the west or from the south or from both these directions. A specific situation is for 2014, for which high concentrations of ragweed pollen appear during the eastern flow. For the western and north-western flow (e.g. years 2009, 2011), the foot print area of air masses covers Germany, northern and central France and the UK. During the southern flow (e.g. 2005, 2006, 2007, 2009, 2011), the foot print area of air masses covers mainly northern and central Italy and Pannonian Plain (Hungary, the Czech Republic, Romania, Slovakia). The main source area of ragweed pollen for the eastern flow is Ukraine.

### Episodes of extremely high airborne ragweed pollen concentration

In the entire period (2005–2014), we analysed the three most severe episodes of high *Ambrosia* concentrations. The HYSPLIT was run here both with the GDAS and WRF data. The dates of episodes are 06–10 September 2005, 11–17 September 2006 and 03–06 September 2014.

#### Episode 1:06–10 September 2005

**Fig. 3:**
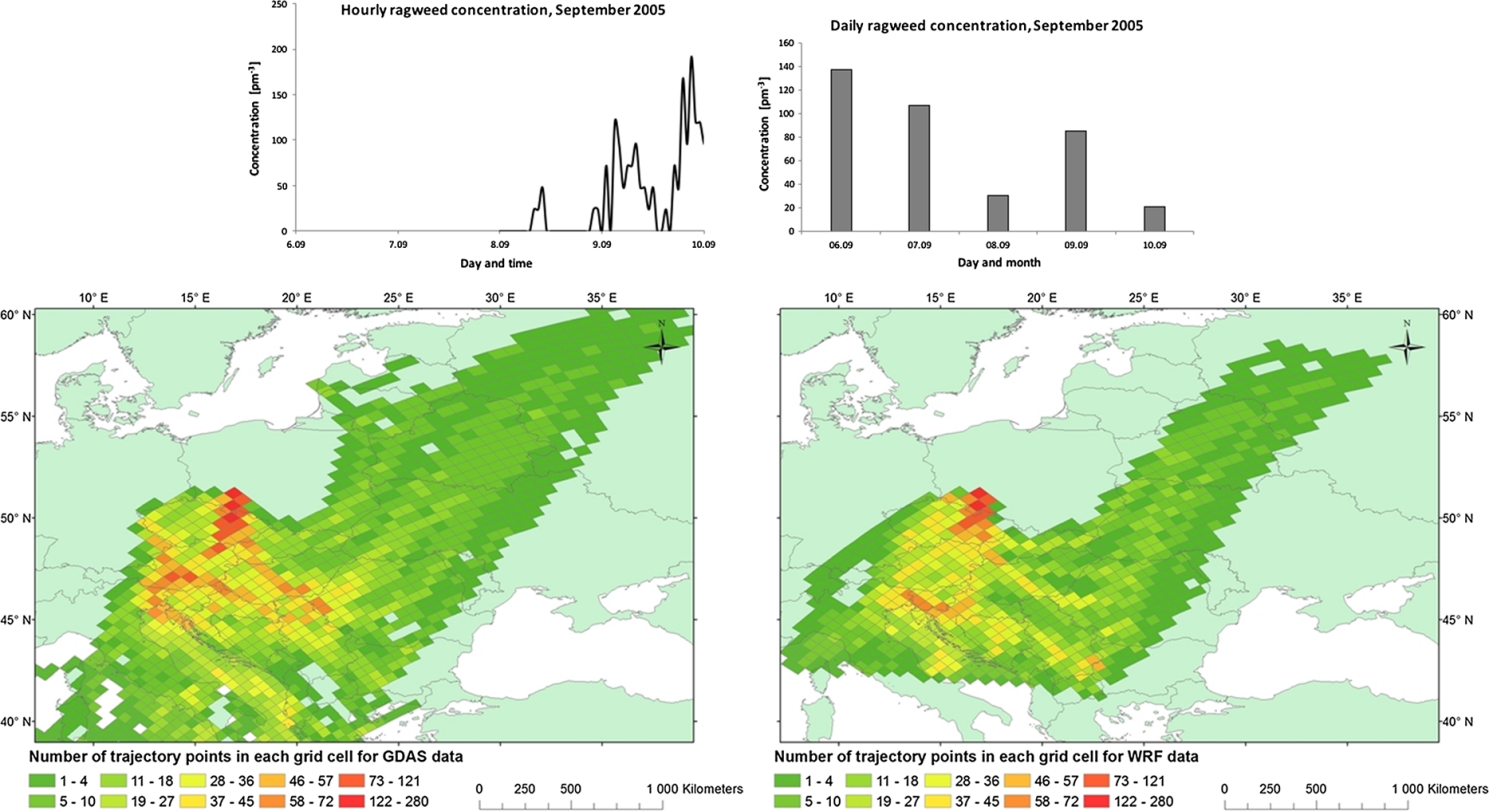
Daily and hourly variation in ragweed pollen counts recorded in the atmosphere of Wrocław 08–10 September 2005 (*upper*). Foot print area of air masses arriving at Wroclaw calculated with HYSPLIT using either WRF (*left*) or GDAS (*right*) input

The highest concentration of ragweed pollen (137 pm^−3^) was reached on the first day of the episode, and this was the highest value observed over the entire 2005–2014 period (Fig. 3). The hourly concentrations of airborne pollen showed two plumes of ragweed arriving early in the morning on the 9th of September and late night and early morning the 10th of September with a peak at 192 pm^−3^ obtained at 9 in the evening the 9th of September. Concentrations of airborne ragweed pollen in the middle of the day were either zero or very low.

The synoptic situation for this episode was influenced by a high-pressure system covering eastern parts of Europe (1020 hPa in the centre of baric system) with a centre over the western part of Ukraine. Within the following 3 days, this system moved towards east. On the 6th of September, the northern and western parts of Europe were affected by a low-pressure system which on 7th of September moved towards east and central Europe, in the place of outgoing high-pressure system. On 9th of September, another high-pressure system was moving from north-western to southern and eastern part of Europe. Wind speed in south-western Poland during 06–10th of September reached up to 5.1 ms^−1^. The dominant direction of advection of air masses is south and south-east. No rainfall was observed for the south-eastern Europe and along the way of air masses towards Poland during 06–09th of September. Rainfall appeared on the 10th of September in the area of south-east Europe (up to 25.6 mm).

The trajectories calculated with the WRF meteorological data (Fig. 3) show that the air masses mainly came from southern directions. The highest frequency of back trajectories is associated with the Balkans region, the Czech Republic and Hungary. Lower frequencies are found over Ukraine, Romania and Italy. The trajectories based on GDAS show a similar picture but also show high numbers over the Alps in Austria and Italy and the Adriatic Sea.

#### Episode 2: 11–17 September 2006

**Fig. 4:**
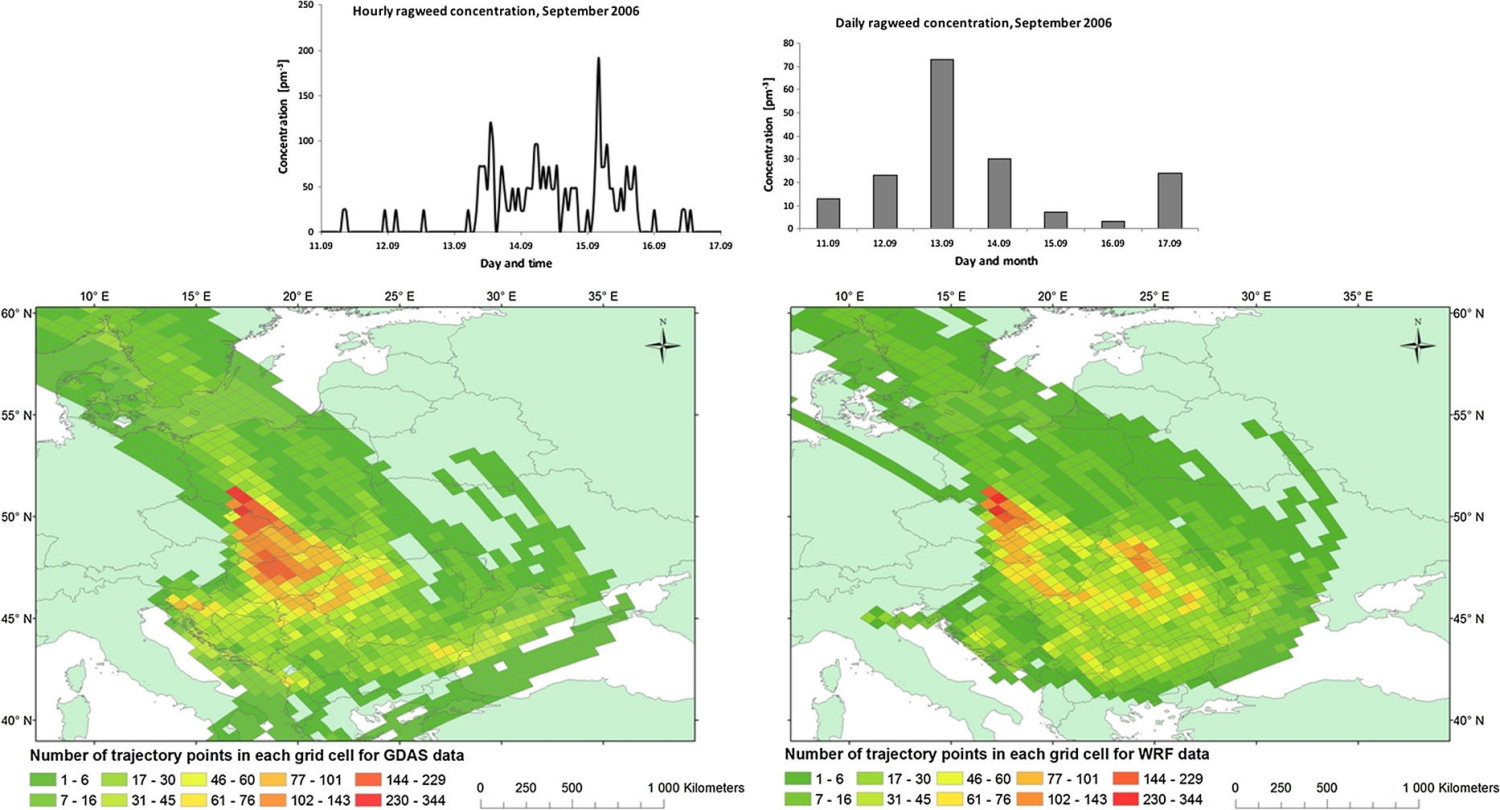
Daily and hourly variation in ragweed pollen counts recorded at Wroclaw 11–17 September 2006 (*upper*). Foot print area of air masses arriving at Wrocław calculated with HYSPLIT using either WRF (*left*) or GDAS (*right*) input

The episode started with low daily mean concentration (13 pm^−3^) which grew up to reach maximum value (73 pm^−3^) on the third day of the episode (Fig. 4). After that, the daily mean airborne pollen concentrations fell down to 3–7 pm^−3^ and increased again on the last day to 23 pm^−3^. The hourly concentrations showed one extended period of airborne ragweed arriving early in the morning on the 13th of September and lasting until the 15th of September at 5 in the afternoon with a peak at 192 pm^−3^ obtained at 4 in the morning on the 15th of September.

For this episode, most of Europe was under a high-pressure system with a centre (1028 hPa) located over central Europe. From 12th of September, this high-pressure system was moving towards eastern part of Europe. The north-western part of Europe on the 11th of September was covered by low-pressure systems, which in the following days moved towards western and central Europe. On the 13th of September, another low-pressure system was moved from western Europe towards north-eastern part of the continent. On the 15th of September, another high-pressure system, from Norwegian Sea, was moving to south-eastern part of Europe and on the 16th of September, its centre was located over north-eastern Europe and reached 1028 hPa. Wind speed in the south-western Poland during 11–15th of September reached up to 5.1 ms^−1^ and increased to 7.1 ms^−1^ on the 16th of September. The dominant directions of air masses advection were south and south-east. No rainfall was observed for the south-eastern Europe and along the way of air masses towards Poland during the entire episode.

The main source of inflow of the air masses observed in Wrocław is area to the south from Poland, in particular the countries on the Pannonian Plain. The highest frequency of trajectories based on both meteorological inputs covers the region of the Czech Republic, Slovenia, Hungary and part of Romania (Fig. 4). The air masses rarely crossed Russia, Ukraine, Italy and Romania. The GDAS data set (Fig. 4) also shows higher values over Slovenia and Croatia and in some of the coastal regions of the Ionian and the Black Sea.

#### Episode 3: 03–06.09.2014

**Fig. 5:**
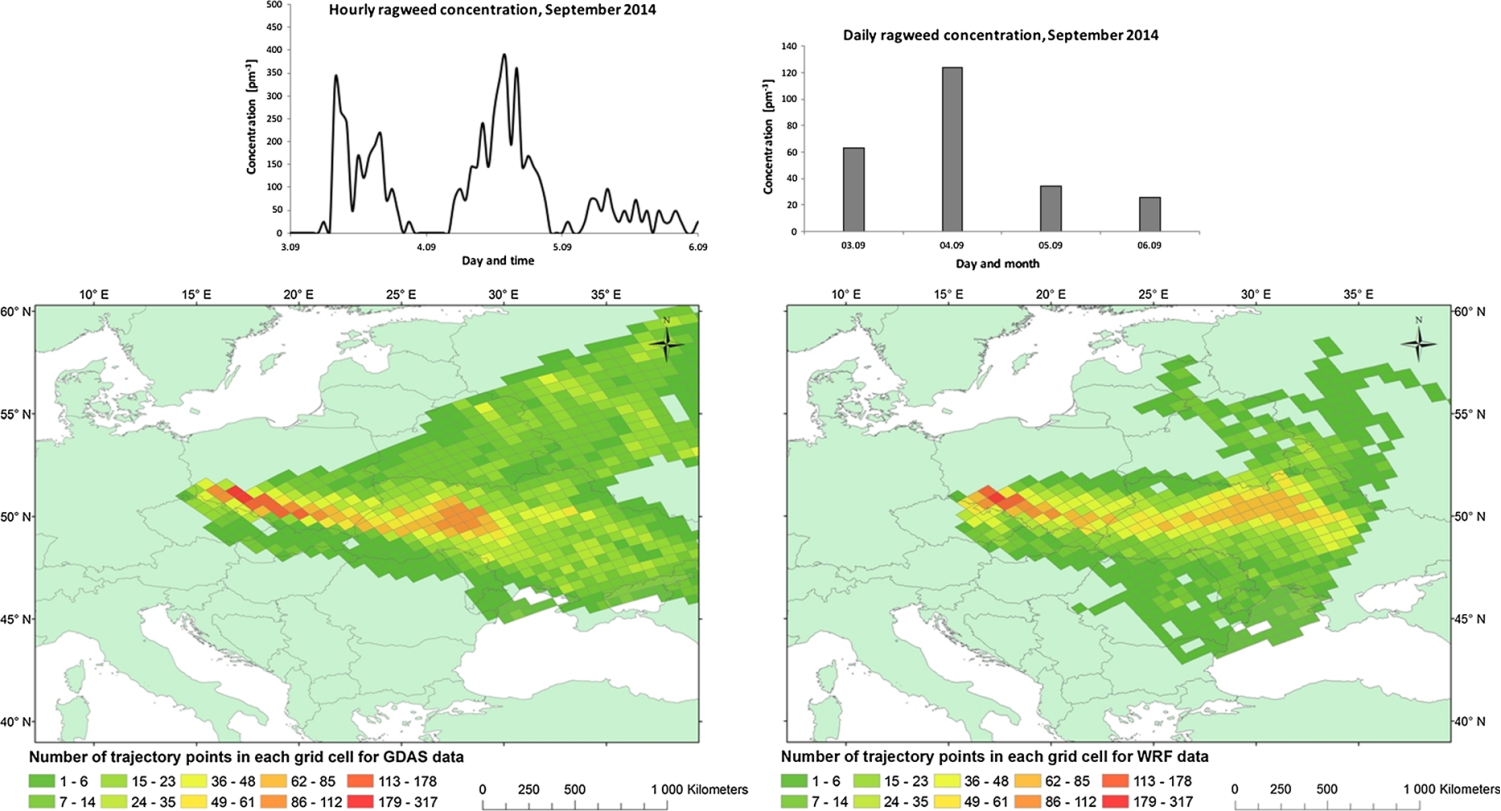
Daily and hourly variation in ragweed pollen counts recorded at Wroclaw 03–06 September 2014 (*upper*). Foot print area of air masses arriving at Wrocław calculated with HYSPLIT using either WRF (*left*) or GDAS (*right*) input

On the first day of the episode, the daily average pollen concentration in the air was 63 pm^−3^, next doubled (124 pm^−3^) and then quickly declined to 34 pm^−3^ on the third day and 26 pm^−3^ on the last day of the episode (Fig. 5). The hourly concentrations of pollen in the air showed two plumes of ragweed arriving early in the morning on the 3rd and the 4th of September with a peak at 384 pm^−3^ measured at 2PM 4th of September. After these two episodes, the concentrations of ragweed pollen were fairly constant between 24 and 48 pm^−3^, both day and night.

The area of north Europe on the 3rd of September was under the influence of a high-pressure system (1028 hPa in the centre located over northern and north-eastern Europe). In following days, until the 06th of September, this system was moving towards south-east over eastern parts of Europe. On the 3rd of September, area of southern Europe was covered by a low-pressure system. This low-pressure system moved to the north and carried warm air masses from eastern Europe towards Poland. During this episode, the advection of air masses was from eastern direction. In the south-western Poland, wind was reaching up to 5.14 ms^−1^. Rainfall was not observed in Poland during this episode, but appeared in southern Europe—up to 102,4 mm on the 6th of September in northern Bulgaria.

General pattern of the frequency of the trajectories, calculated with WRF and GDAS meteorology, is similar (Fig. 5). Both HYSPLIT model runs suggest south-eastern part of Poland, eastern part of the Czech Republic, Ukraine, Moldova as source regions of air masses. The WRF model run shows increased frequencies of trajectories also for Romania and small area of Belarus and Russia. The GDAS trajectories map covers Belarus and a large part of Russia too (Fig. 5). The source area of ragweed pollen was eastern parts of Ukraine.

## Discussion and conclusion

Our results show that ragweed pollen during 2005–2014 was not common in the air of Wrocław, and its concentration is generally low during the flowering season. The exceptions were high pollen episodes, which were connected with flow of air masses from south (years: 2005, 2006, 2007, 2009 and 2011) or west Europe (years: 2009, 2011). In 2014, a high airborne pollen episode was related to the eastern flow, with eastern Ukraine being likely source area. The study confirmed the existence of known ragweed centres but has also shown that other ragweed centres near Wrocław were not present. High episodes were observed almost every year and the hourly observations showed that the episodes are observed both at daytime and at nighttime. Very short episodes with high airborne pollen concentrations contribute with the majority of the records to the total 2-month sum of the average daily ragweed pollen concentration for the individual years. For 1 year (2005), this contribution was 96%. This confirms that outside the main ragweed centres high concentrations of ragweed pollen are episodic. This finding is supported by previous studies in Poland (Smith et al. 2008; Kasprzyk et al. 2011), Turkey (Zemmer et al. 2012), Denmark (Sommer et al. 2015), UK and the Netherlands (de Weger et al. 2016).

The figure with the number of trajectory crossing each grid cell for August and September (Fig. 2) is interesting with respect to the increased knowledge on mapping ragweed distribution in Europe. Comparing the footprint area of air masses for high days (Fig. 2, right) with the air masses incoming during entire investigated ragweed season (Fig. 2, left) shows that on the high days the air masses always come from the eastern part of the Pannonian Plain or eastern Ukraine. Both areas have previously been identified as major ragweed source areas (Smith et al. 2013). Analysis of high airborne pollen days for individual years (Fig. S2, e.g. year 2006 and 2008) confirms influence of two other known ragweed centres in Europe: France (Thibaudon et al. 2014) and northern Italy (Bonini et al. 2015).

On the low days, air masses come from many different directions, but low frequencies are observed, e.g. for the Pannonian Plain (Fig. 2). This suggests that if air masses originate from the Pannonian Plain during the main flowering season, then there is a large risk of high airborne ragweed pollen concentrations in Wrocław. Similar, the combined result from the figures suggests that there are no strong ragweed populations in the vicinity of Wrocław as this expectedly would cause episodes not originating from the main ragweed centres. Most likely the low ragweed pollen concentrations were due to either a few and scattered populations in Poland or Germany or due to pollen grains that have stayed airborne for a long time. A previous study by Cunze et al. (2013) on presence/absence of ragweed pollen showed a very little presence in the south-west Poland. Our study confirms these findings and also suggests that the abundance of ragweed in south-western Poland is limited. This information is important both in relation to the forecasting of ragweed pollen concentrations in the air but also in the development of mitigation strategies as effective strategies vary with the distribution and abundance of ragweed plants.

The results in Figs. 3, 4 and 5 are particularly interesting with respect to air mass flow from the main centres in both Ukraine and the Pannonian Plain. Firstly, the difference between the two set of model calculations shows that only the WRF-HYSPLIT simulations are affected by the Tatra Mountains (Poland—Slovakia border). The differences are not large, in terms of the total trajectory number of trajectories shifted, but may bring important new information as the Tatra Mountains have been previously identified as an important factor in understanding of ragweed dispersion into Poland (Smith et al. 2008). Both Figs. 3 and 4 show that the WRF-HYSPLIT simulations suggest that the major source areas are directly in the western part of the Pannonian Plain and with limited contribution of air masses inflow from the eastern part. This matches well with existing knowledge on ragweed inventories that were developed for both the Pannonian Plain (Skjøth et al. 2010) and Austria (Karrer et al. 2015). The GDAS-HYSPLIT picture is, however, less clear. These calculations indicate the Pannonian Plain as a source, but also part of the Balkan region is suggested as potential areas. This area is currently considered to have limited ragweed infection (Šikoparija et al. 2009; Karrer et al. 2015). Similar unclear picture using the GDAS-HYSPLIT setup was recently found by de Weger et al. (2016) in analysing the Rhone valley as a potential source region for ragweed. The most likely cause to our difference between GDAS-HYSPLIT and WRF-HYSPLIT is that the trajectories based on the WRF calculations—due to the higher spatial resolution—have a better description of the relief of the landscape in the main ragweed regions.

Figure 5 shows a difference in the air mass flow towards Wrocław. The WRF-HYSPLIT simulations show a minor contribution of air masses inflow from the Pannonian Plain, and the major is due to a direct flow of air masses from Ukraine. These air masses experienced limited dispersion during the transport over the Carpathian Mountains and eastern Poland. The HYSPLIT calculations that are based on the GDAS data set show a diverse picture and do not only indicate eastern Ukraine as a foot print area of air masses but also areas of central Russia. In contrast, the HYSPLIT calculations based on WRF show mainly a contribution of air masses inflow from the area of eastern Ukraine and a minor contribution from eastern part of Pannonian Plain. This matches well with existing knowledge on main ragweed centres in Europe. Assuming that the 2014 episode is similar to previous episodes from Ukraine (Kasprzyk et al. 2011), then this highlights the importance on having detailed meteorological data set for ragweed dispersion models that covers all of Europe including Ukraine. The results also suggest that improvements with HYSPLIT can be obtained by replacing the GDAS data set with high-resolution data as the air mass contribution from Belarus and western parts of Russia in the GDAS data set is less likely to have contributed with substantial ragweed pollen concentrations in Wrocław. Belarus has previously not been identified as a ragweed centre. In fact, these two areas are dominated by forest cover (Skjøth et al. 2008)—thus not considered a favourable ragweed habitat. The difference on the HYSPLIT results between the two meteorological data sets therefore confirms previous studies on olive pollen (Hernandez-Ceballos et al. 2014) that the input data to HYSPLIT are an important parameter to consider in aerobiological studies.

The main findings of this study are that foot print studies on ragweed benefit from high-resolution meteorological data sets. The Pannonian Plain, Ukraine, France and northern Italy are the source areas of pollen which cause episodes of high ragweed pollen concentration in the air over south-western Poland. This information is important in relation to forecasting, constructing inventories and in designing mitigation strategies on ragweed.

## Electronic supplementary material

Below is the link to the electronic supplementary material.

Fig. S1 Number of trajectory crossing each grid cell for August and September for low values of ragweed concentrations for years 2005–2014. Calculation are based on HYSPLIT using GDAS data and 96-h back trajectories for Wrocław at 500, 1000 and 1500 m (TIFF 3377 kb)

Fig. S2 Number of trajectory crossing each grid cell for August and September for all (*left*) and high values (*right*) of ragweed concentrations for individual years 2005–2014 (There were no high values observed in 2010 and 2013). Calculations are based on HYSPLIT using GDAS data and 96-h back trajectories for Wrocław at 500, 1000 and 1500 m (JPEG 27698 kb)
