# Supplementary figures and images for: Source regions of ragweed pollen arriving in south-western Poland and the influence of meteorological data on the HYSPLIT model results
Source: Aerobiologia (Bologna). 2017 Jan 20;33(3):315–26. doi: 10.1007/s10453-017-9471-9 (PMC5591811; doi:10.1007/s10453-017-9471-9)

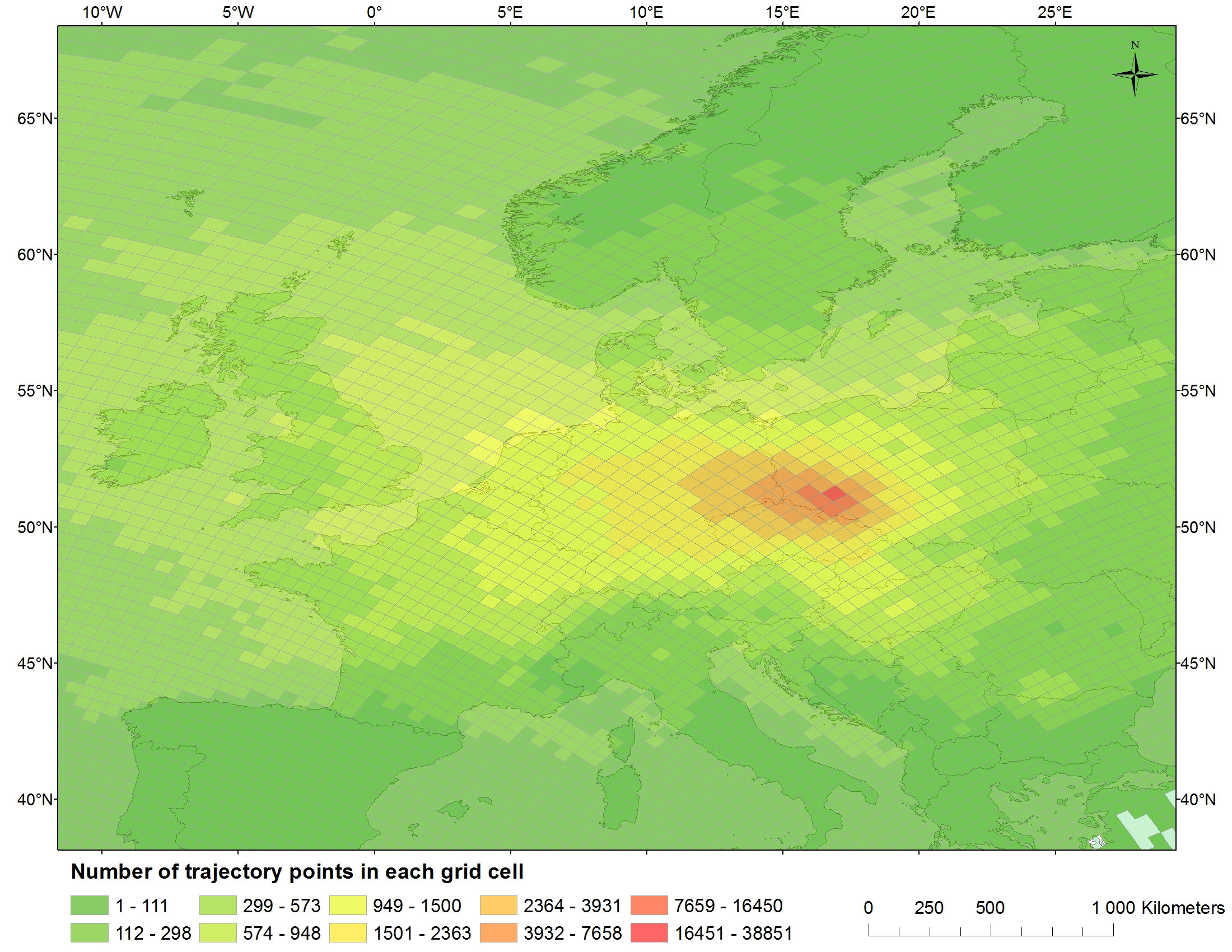

Supplement: Supplementary file 1 — Number of trajectory crossing each grid cell for August and September for low values of ragweed concentrations for years 2005–2014. Calculation are based on HYSPLIT using GDAS data and 96-h back trajectories for Wrocław at 500, 1000 and 1500 m (TIFF 3377 kb) [file 10453_2017_9471_MOESM1_ESM.tif]

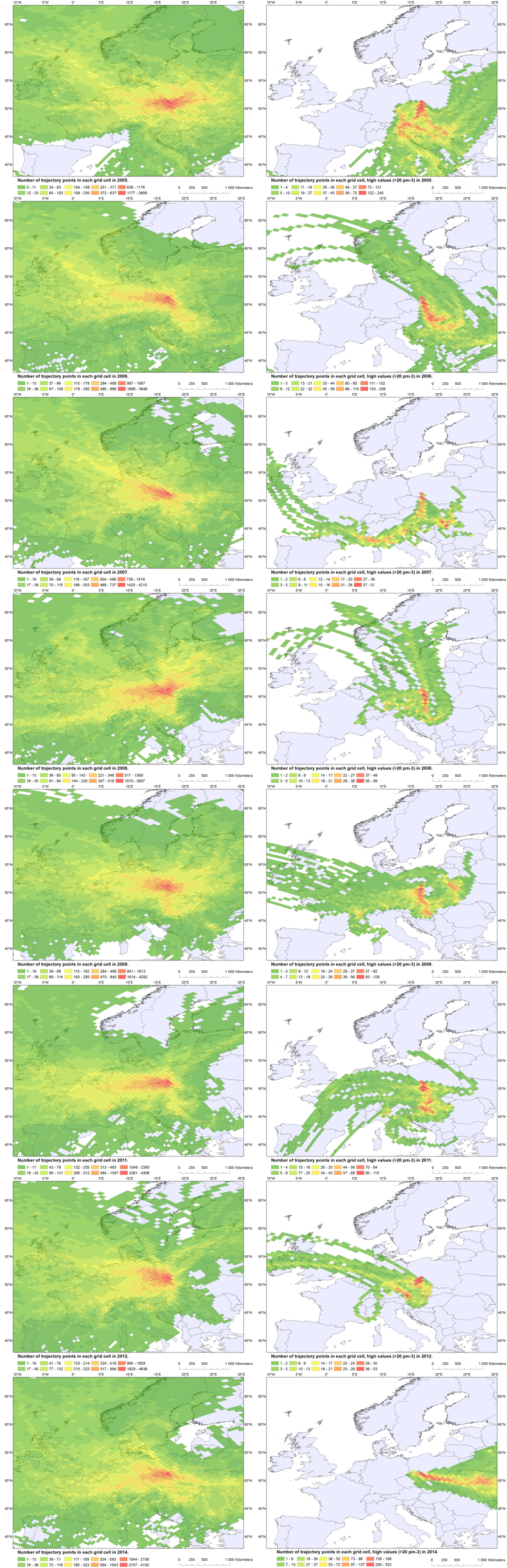

Supplement: Supplementary file 2 — Number of trajectory crossing each grid cell for August and September for all (left) and high values (right) of ragweed concentrations for individual years 2005–2014 (There were no high values observed in 2010 and 2013). Calculations are based on HYSPLIT using GDAS data and 96-h back trajectories for Wrocław at 500, 1000 and 1500 m (JPEG 27698 kb) [file 10453_2017_9471_MOESM2_ESM.jpg]
